# Precision medicine: affording the successes of science

**DOI:** 10.1038/s41698-022-00343-y

**Published:** 2023-01-04

**Authors:** Christine Y. Lu, Vera Terry, David M. Thomas

**Affiliations:** 1 Department of Population Medicine, Harvard Medical School and Harvard Pilgrim Health Care Institute, Boston, MA USA; 2 Omico: Australian Geneomic Cancer Medicine Centre Ltd, Darlinghurst, Australia; 3 Genomic Cancer Medicine Laboratory, Garvan Institute of Medical Research, Omico: Australian Genomic Cancer Medicine Centre Ltd, St Vincent’s Clinical School, Faculty of Medicine, UNSW, Sydney, Australia

**Keywords:** Drug development, Molecular medicine

## Abstract

Science has made remarkable advances in understanding the molecular basis of disease, generating new and effective rationally-designed treatments at an accelerating rate. Ironically, the successes of science is creating a crisis in the affordability of equitable health care. The COVID-19 pandemic underscores both the value of science in health care, and the apparently inevitable tension between health and the economy. Drug development in ever-smaller target populations is a critical component of the rising costs of care. For structural and historical reasons, drug development is inefficient and poorly integrated across the public and private sectors. We postulate an alternative, integrated model in which governments and industry share the risks and benefits of drug development. The Australian government recently announced support for a AU$185 million innovative multi-stakeholder public-private partnership model for sustainable precision oncology, accelerating biomarker-dependent drug development through integrating clinical trials into the standard of care.

## Introduction

Scientific advances in understanding the molecular basis of diseases are generating new and effective rationally-designed treatments that promise to improve health outcomes. However, health systems face increasing challenges to the sustainability of equitable health care. Even prior to the COVID-19 pandemic, health expenditure as a fraction of gross domestic product (GDP) has been rising year-on-year in all higher-income countries, reaching 17% in the US and 10.2% in Australia in 2019. Costs are rising in part due to the demand for health care by growing and ageing populations. Drug development is a major contributor to health costs – the average cost of getting a new drug into the market is estimated at US$1.3 billion^1^. Countries such as the US, the UK, and Australia are tackling the affordability crisis by engaging in risk-sharing agreements with industry (also called managed entry agreements, patient access schemes, among other terms)^2^. Globally, governments and industry recognize that fundamental changes are essential to help address the sustainability of health care.

This issue is particularly pressing for cancer, the leading cause of death in higher-income countries. Fundamentally a genetic disease, cancer treatment is being radically transformed by the twin influences of genomic technologies and rational drug development. By 2018, biomarker-dependent drugs accounted for 42% of approvals by the US Food and Drug Administration (FDA) in 2018, a doubling from 2014^3^. The widening gap between the potential of science to improve health, and its affordability, is exemplified by non-small cell lung cancer, the leading cause of cancer deaths world-wide. There are now 10 molecularly distinct subtypes of non-small cell lung cancer for which there are effective therapies, accounting for more than 50% of all affected patients^4^. However, only 3 of these therapies are publicly reimbursed in Australia. The Australian Pharmaceutical Benefits Advisory Committee considers five major factors in their recommendations for new drug coverage and reimbursement^5^. Clinical impact comprises only one of these five factors, with the remainder concerning cost-effectiveness and budgetary impact.

In March 2022, the Australian government announced support for a radical approach to solving this problem, the establishment of a AU$185 million (US$130 million) innovative multi-stakeholder public-private joint venture for precision oncology^6^, whose conceptual framework is outlined in this article. We explore the current inefficiencies in drug development in single-payer health systems (exemplified by Canada, Denmark, Norway, Australia, Taiwan and Sweden) due to the often adversarial relationship between governments and industry. Further, we propose that affordable patient access to health care is achievable through collaborative engagement between governments and industry in drug development. Although focused on precision oncology, the ideas outlined here are broadly relevant to the sustainability of science-led transformations in health care.

## Drug development: challenges and opportunities

**Fig. 1:**
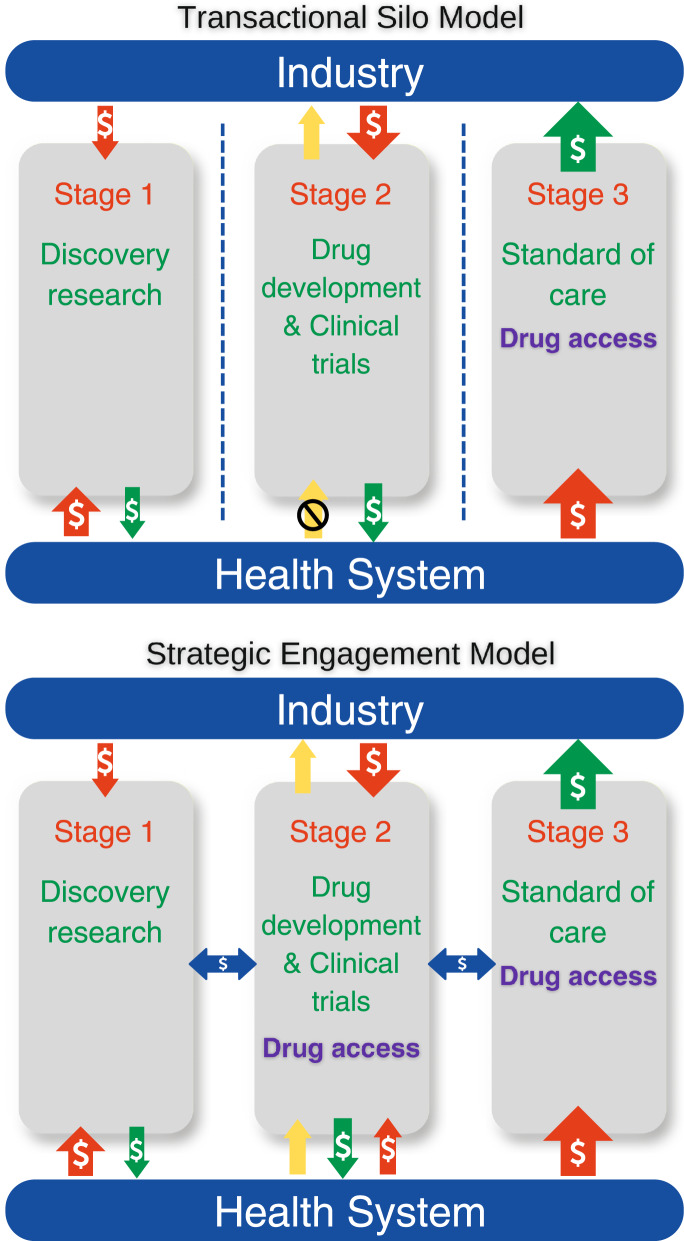
Three stages of drug development in the existing (transaction silo) model vs. proposed collaborative (strategic engagement) model for improving cost-efficiency and sustainability of innovative health care. Red arrows depict investments; green arrows depict returns; blue arrows indicate exchanges of value; yellow arrows indicate information transfer. Sizes of arrows represent the relative magnitude of monetary value transfer.

Public and private sector engagement in drug development can be conceptualized in three distinct stages along the value chain^7^ (Fig. 1). Stage 1 involves discovery research – predominantly funded by government and philanthropy, and undertaken in the public sector. Basic medical research generates intellectual property, which is licensed or sold by academic institutions to industry, partially recouping the costs of research. Stage 2 involves the industry pursuing priority drug targets with a focus on lead compound identification and medicinal chemistry, followed by clinical trials (phases 1 to 3). Industry funds the health sector to conduct trials in increasingly biomarker-selected populations, often within hospitals. The failure rate is high, with 15-35% of drugs reaching phase 2 trials ultimately obtaining regulatory approval^1^. This figure is lower for oncology at 6.7%. In stage 3, new drugs may become a reimbursed standard of care after policymakers review for clinical utility and cost-effectiveness in certain jurisdictions. In single-payer health systems, the public sector pays industry for these drugs, with the price in reflecting the total costs of drug development (including those that fail), as well as maximizing commercial returns.

This model is characterized by siloing of each stage from the other, and the use of money as the unit of value transfer. Each partner pays for the services or goods with the goal of maximizing monetary gains within each stage, and the cumulative cost rises as drug development proceeds from stage 1 to stage 3. Considering the interdependent nature of drug development, we contend that the lack of integration between health sector and industry contributes to inefficiencies that increase the net cost.

Stage 2, focussing on clinical trials, is arguably where the greatest interdependence between the public and private sectors lies. Much of the total cost of drug development is due to clinical trials^8^, which are necessarily conducted by industry in the context of health systems. Through increasingly complex regulatory and governance processes, health systems contribute to trials inefficiency and costs^9–12^. Clinical trials are not considered core business for most health systems. For example, only 8% of adult cancer patients participate in trials in Australia^13^. While money is the major unit of value exchange between industry and health systems, each party has additional assets of mutual interest that could form an alternative basis of value exchange. The health system’s main assets are the patients required for clinical trials and the data that is a natural byproduct of health care delivery. The value of this data is currently largely unrealized, although it constitutes the raw substrate for rational drug development and health technology assessment. Industry expends billions on the creation of ‘real-world data’ assets^14^, exemplified by the market capitalization of entities like Tempus and Flatiron Health^15,16^. Ironically, industry must ultimately recoup the additional costs of generating real-world data from the health system as part of the total costs of drug development.

Industry’s main asset of interest to the health system are the drugs and other technologies it produces to improve health outcomes, currently mostly accessible universally only in Stage 3. For industry, the costs of manufacturing drugs are a fraction of the total costs of trials (patient screening, consent, data collection). Nonethless, increasing participation in biomarker-dependent clinical trials would accelerate drug development.

Can we conceive a public-private partnership in which the health system actively supports clinical trials as a standard of care, effectively realizing the value of information in exchange for early drug access, while increasing the overall efficiency and decreasing the costs of drug development?

## Clinical trials: a new standard of care?

These concepts have clear application in oncology, where advances in genomics have radically accelerated drug development. It is estimated that, of more than 840 oncology drugs in development in 2018, >90% are biomarker-dependent^17^. A biomarker, as defined by FDA and the National Institutes of Health, is a “characteristic that is measured as an indicator of normal biological processes, pathogenic processes, or responses to an exposure or intervention, including therapeutic interventions”^18^. As noted above, clinical trials are not a core part of standard oncology practice. In the 1990s, a phase 1 study of a new chemotherapy offered a low response rate (e.g., 5%) with unquantifiable risk of toxicities. The situation is changing rapidly. Participation in a phase 1 study of a rationally-designed drug directed at a matched biomarker now offers a response rate in excess of 30%^19^. Consider doxorubicin, a standard of care for a patient with newly diagnosed metastatic sarcoma, with response rates of between 10% and 30%^20–22^. For patients carrying the relevant biomarker, a phase 1 study today appears to offer a better chance of response than the standard of care.

It is important to note that trials-based therapies carry inherently greater uncertainty than standard-of-care treatment, which should be considered in the following proposed model. For this reason, we have focused on the terminal stages of a cancer patient journey, where most patients run out of standard-of-care options. In these circumstances of unmet medical needs, clinical trials of biomarker-dependent therapies offer additional important options for patients who remain fit enough for treatment. Further, we anticipate that trial drugs may have roles even at earlier stages of the cancer journey, where standard-of-care treatments exist. Participation ethically approved clinical trials randomizing new against standard-of-care cancer treatments could offer access to promising new therapies. Given the uncertainty attached to the clinical benefits of participation in clinical trials, it will be important to monitor clinical outcomes prospectively as part of the proposed model.

## Enhanced efficiency and reduced costs of biomarker-dependent drug development

**Fig. 2:**
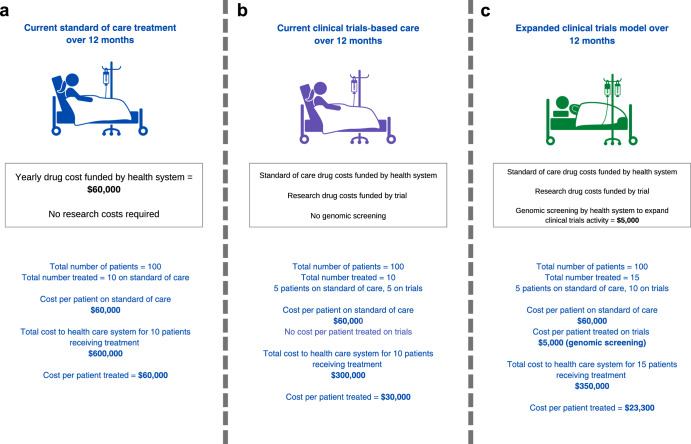
Genomic screening and cancer treatment in health systems and clinical trial cost-offsets. ©[Leremy Gan] via Canva.com. **a**: current standard of care treatment; **b**: clinical trials-based care; **c**: expanded clinical trials supported by population-level screening.

If the principle of patient benefit is accepted, does increased access to clinical trials make economic sense? The rising costs of new drugs has fundamentally changed the answer to this question. Let us assume that the health system bears standard-of-care treatment cost of $60,000 per patient per year, such that the total cost to the health system is $600,000 for 10 patients treated (Fig. 2a). If 5 of 10 patients access a drug provided for free by an industry partner through a trial, this provides a treatment cost-offset of $300,000 to the health system compared to standard-of-care drug access (Fig. 2b). Effectively, a fraction of the government pharmaceutical budget could be partially re-purposed to support the expansion of trials as a standard of care. Even if the health system was to invest an additional $5,000 per patient to support genomic screening and participation in clinical trials and cost per patient treated is $23,300, the system would still save $36,700 per patient assuming an additional 5 people would enrol and receive treatment through trials (Fig. 2c; compared to Fig. 2a, cost per patient treated is $60,000). The more people access therapies via clinical trials, the better for the economic benefit to the health system. To incentivize industry to conduct trials, the health system needs to contribute to making trials more efficient. For industry, an efficient increase in clinical trials participation reduces the length of time to conduct trials and associated costs.

For oncology drug development, health system-industry collaboration offers many opportunities for efficiencies and cost savings. Oncology drugs increasingly target biomarkers, often in the form of mutations detectable by genomic screening. Cancers are being increasingly subdivided therapeutically according to these biomarkers, generating the need to identify specific subpopulations carrying the cognate therapeutic biomarker for clinical trials. The cost of screening large numbers of patients to find these subgroups adds to the costs of drug development. A relevant drug target may be present in fewer than 1% of the general population, meaning that 100 people need to be screened to find one eligible participant. Currently, industry bears the costs of identifying such patients for clinical trials through diagnostic screening tests, almost invariably focusing on a single gene target, conducted separately for each trial and participating institution. Typically these single gene tests reflect the regulatory bodies’ requirement that a purpose-built companion diagnostic be approved with the therapy – a co-dependent technology.

The advent of comprehensive genomic profiling (CGP) can radically transform the efficiency of identifying subpopulations for clinical trials. CGP enables the screening of hundreds of potential drug targets in a single assay^23,24^, such that one test could be used to triage patients for dozens of trials. Realising the efficiencies of this approach requires the shift from trial-specific single gene testing, to CGP screening of patient populations on behalf of multiple trials.

**Fig. 3:**
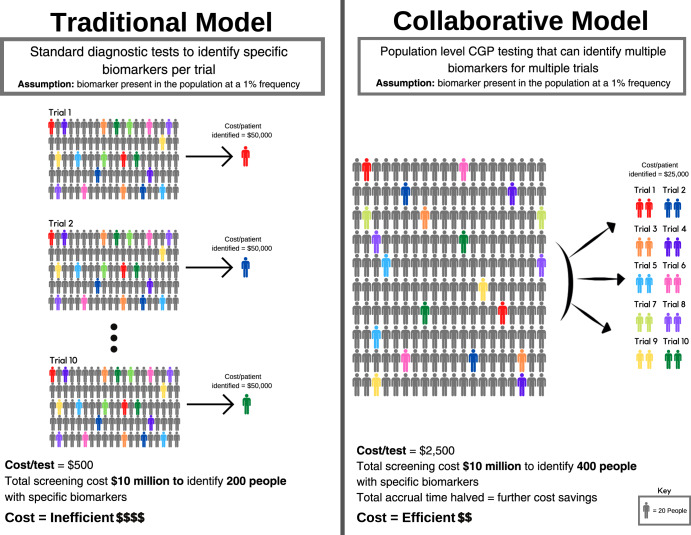
Efficient identification of eligible patients for biomarker-dependent drug trials by population screening. ©[Pixeden] via Canva.com.

To illustrate this, consider the following hypothetical example of 10 companies intending to perform 10 trials (Fig. 3). Each trial is dependent on screening for a distinct biomarker present in the population at a 1% frequency. Each trial needs to screen 2,000 patients to identify 20 patients with the relevant biomarker (assuming for the purposes of argument 100% enrolment of suitable candidates onto each trial). Using a diagnostic screening test that costs $500 per individual, a 20-patient trial requires a $1 million screening budget (Fig. 3a). For a trial that costs $1 million to run (20 patients x $50,000 per patient enrolled), screening may account for half of the total cost. Collectively, 10 trials need to screen 20,000 patients using 10 biomarker-specific and purpose-built tests, with total screening expenditure of $10 million.

However, if the 10 biomarkers for these trials are mutually exclusive, then in principle only 2000 patients need to be screened to support all 10 trials through a single CGP test (Fig. 3b). If a CGP test costs $2500 per individual, screening 2000 individuals would cost $5 million to support 10 trials. Alternatively, using the original $10 million budget for all 10 trials, 4000 patients could be screened, identifying those patients twice as fast, thereby cutting the time to trial completion by half. In summary, the reduced costs of identifying eligible patients, and the acceleration of trials completion, are the twin drivers for decreasing the costs of drug development. Furthermore, CGP cost is declining, making population-level screening for guiding drug treatments more feasible.

Screening for 10 trials involving multiple industry partners, requires an ‘honest broker’ operating on behalf of all parties, with access to the patient populations to be screened. For these reasons, health systems, either directly or via an ‘honest broker’, are ideally placed to undertake biomarker screening in partnership with industry. The funding for CGP tests may be cost-efficient for health systems as patients might shift from system-reimbursed therapy to trial-based therapy.

**Fig. 4:**
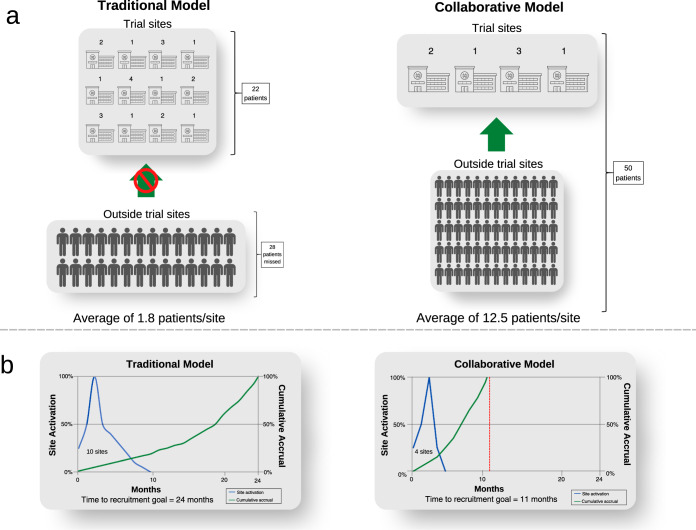
Increased trial efficiency due to shift from site-based to population screening. **a** Screening and trial sites for clinical trials by model. The numbers above each institution represent eligible patients for a biomarker dependent trial. **b** Differences in efficiency for trial recruitment between models. ©[Visual Generation, Pixeden] via Canva.com.

The collaborative model has an important additional benefit by expanding screening from sites where trials are conducted, to a much larger population across the entire health system (Fig. 4). Screening is limited to trial sites in the traditional model. The sponsor needs to open more trial sites to maximise the population to be screened because of the patient catchment area of the institution. If sites do not routinely undertake screening, this is funded by each trial. Opening each trial site adds costs and time related to governance and monitoring complexities. In contrast, the collaborative model would need fewer trial sites since the number of trial sites is predicated on the site trial capacity, not its patient catchment. To illustrate this point, for rare cancer populations, trials may not be feasible if the sponsor opens trial sites at 12 institutions to identify 22 patients for a trial, missing the opportunity of recruiting 28 people who are outside the trial sites (Fig. 4a). On the other hand, all 50 people could be identified by an independent population-based screening program in the collaborative model, who are referred to only four sites opened for trial conduct, increasing trial efficiency (Fig. 4b). This increases the clinical experience at each site, and the contribution of site investigators in answering the trial question. It also increases the system capacity for trials in total, by distributing the burden of trial conduct across a broader range of treating centres, whereas currently the burden of trials falls asymmetrically on high-volume centres, whose capacity may be saturated. Finally, it increases clinical trials engagement of centres which might otherwise not participate due to their patient volumes. Some of the cost savings outlined above could be reallocated to subsidies to support patient travel across a broader network of trial centres.

Importantly, the collaborative model will lead to better health outcomes for patients overall, for several reasons: (1) the total number of patients receiving biomarker-dependent therapies should increase compared with the existing model, due to enhanced access to trials; (2) the greater efficiency of CGP screening means that a greater fraction of patients carrying the relevant treatable biomarker will be identified than is currently the case; and (3) the greater speed and efficiency of trials conduct will reduce the net costs of drug development. In short, the greater the number of trials, the greater the amortization of the costs of CGP screening (or the greater the numbers of patients that can be screened with the same resource).

## Structural considerations

The interface between health systems and industry must take into account several considerations. Health systems are not well-designed for direct engagement with industry. The entrepreneurial approach important to a successful partnership with industry is not a common feature of bureaucracies. To date, precision medicine has typically been funded using relatively short-term, project-based research funding, which are usually limited in scale and do not consider long-term sustainability. Moreover, there are good reasons to retain some degree of independence of government from industry, recognizing the competing interests between the economic, health, citizen protection and regulatory functions of government in drug development. Finally, in some health systems, there is variable alignment between federal and state or provincial governments. In Australia, although precision medicine constitutes a matter of national interest, it is delivered through hospital networks primarily funded by state/territory governments. A similar model also exists in Canada. Integrating the interests and roles of both federal and state/territory governments has been a structural impediment to nationally consistent health care.

A solution to these requirements is being tested in Australia is the creation of a non-profit company (Omico) with both state and Federal government funding, which has developed a joint venture framework for co-investment with industry in a national precision oncology platform^6^. Omico provides a stable, national ‘honest broker’ role on behalf of the health system with the cultural and legal freedom to develop innovative, collaborative engagement between industry and the health system. In practice, Omico commissions biomarker screening for referred patients, and returns a report to the referring clinician, who then decides whether the recommended clinical trial is appropriate for their patient. We note that the proposed collaborative model has application beyond cancer to all health conditions relying on biomarker-dependent drug development (e.g., cardiovascular, renal, endocrine).

## Economic benefits

In addition to better health care, there are economic benefits to greater engagement between the public sector and industry. Health is typically seen as an ‘expenditure’ portfolio for the government. The pharmaceutical industry generates revenue in excess of US$1.25 trillion globally, growing at 5% per annum^25^. For a population of 25 million, the Australian pharmaceutical industry contributes more than AU$8.9 billion (US$6.3 billion) annually to the economy, and supports almost 23,000 full-time jobs^26^. Cancer clinical trials alone contribute over AU$1 billion (US$0.7 billion) annually to the Australian economy, supporting nearly 7,000 highly skilled jobs^26^. Investments in the life sciences sector generates economic growth, jobs in education, training, infrastructure and support roles, stimulating commercialization of medical research through accelerated local biomarker-dependent drug development, and enhancing greater engagement with global pharmaceutical industry. These outcomes constitute strategic goals common to the health system and economy, the academic sector, and industry.

There are obvious challenges to the success of public-private partnerships that include social and legal issues, political will, bureaucratic inertia, and legitimate competing interests. Social and legal issues relate to privacy concerns and commercialisation of health data. This is partly due to the perception of antagonistic interests of industry and citizens, and partly due to perceptions of research as irrelevant to health care. Disengagement of the public sector from industry leaves patients without access to drugs, and society with fewer options for economic growth. At the other extreme, a fully privatised healthcare model may compromise long-term societal value. Tempo also contributes to the challenges: the low risk appetite of health systems leads to structural and cultural conservativism, while research has an inherently greater risk appetite. The question is whether the risk appetite of health systems can be adjusted to benefit patients who are dying from treatable diseases.

## Future directions

The model proposed ambitiously advocates for clinical trials as a standard of care. Due to the inherent clinical and economic uncertainties, we have proposed that it should be implemented initially for patients who have exhausted conventional treatment options. However, the model could apply at all stages of the cancer journey, where participation in randomised clinical trials could be a standard-of-care. This approach would require sufficient evidence from earlier phase testing to ethically justify randomisation between existing and new treatments. Biomarker screening is also evolving rapidly. Beyond comprehensive genomic panels, whole genome and transcriptome sequencing approaches are being evaluated, and offer potential advantages^27^. While routine pathology processing and costs make focused panels more practical currently, the technology is rapidly evolving and costs of whole genome sequencing are falling, and routine practice will likely adapt over time. One more general benefit of integration of research into standard-of-care is to adapt conservative health systems to align better with the accelerating pace of scientific development. Notably, there are therapeutic biomarkers beyond genomics (DNA and RNA), including proteomics and the microbiome, that are transforming cancer clinical research and care. At the system level, a data-driven approach is clearly important to optimising the integration of research into standard-of-care effectively, equitably, and sustainably.

The increasing faction of GDP due to health expenditure world-wide is driven by the inexhaustible social appetite for better health outcomes and the successes of science. Recent experience with COVID-19 and HIV have shown that science is critical to solving health crises in real-time, and reinforced the tension between health and the economy. The crisis in long-term affordability of health care is exacerbated by aging populations and relative diminution of taxpayer base funding health care. Two conclusions are clear: (1) the rate-limiting step in realising these outcomes is no longer scientific, but lies in our health system’s ability to integrate science into health care; and (2) the public and private sectors have complementary roles in delivering the benefits of science to mankind. Like climate change, health care is too important to society to be left either to the public or private sectors alone, and can only be addressed in partnership.

## Data Availability

not relevant.

## Code availability

not relevant.
